# Trends in Repair vs. Biceps Tenodesis for Superior Labrum From Anterior to Posterior (SLAP) Tear: An Epidemiological Study

**DOI:** 10.7759/cureus.27096

**Published:** 2022-07-21

**Authors:** Ian S Hong, Joshua D Meade, Bradley L Young, Ziqing Yu, David P Trofa, James E Fleischli, Nady Hamid, Dana Piasecki, Bryan M Saltzman

**Affiliations:** 1 Orthopedics, Musculoskeletal Institute, Atrium Health, Charlotte, USA; 2 Orthopedic Surgery, Musculoskeletal Institute, Atrium Health, Charlotte, USA; 3 Orthopedic Surgery, Columbia University Irving Medical Center, New York City, USA; 4 Sports Medicine Center, OrthoCarolina, Charlotte, USA

**Keywords:** epidemiology, slap repair, repair, slap tear, biceps tenodesis

## Abstract

Background

The purpose of this epidemiologic study was to report general trends in the number of superior labrum from anterior to posterior (SLAP) tear repairs and biceps tenodesis performed along with the patient and hospital characteristics within the period of 2016-2018.

Methods

National Ambulatory Surgery Sample (NASS) database was used as the source of data for this epidemiologic study of the United States population. Current Procedural Terminology (CPT) codes were utilized to identify patients that underwent SLAP repair or biceps tenodesis between 2016 and 2018.

Results

The national estimates of encounters from the NASS database reported 29,931 SLAP repairs in 2016 and each subsequent year saw a decline to 26,509 repairs in 2017 and 23,451 repairs in 2018 (p<0.0001). Conversely, in 2016 there were 19,221 biceps tenodeses and each subsequent year saw an increase to 21,625 biceps tenodeses in 2017 and 22,867 biceps tenodeses in 2018 (p<0.0001).

Conclusion

The results of our epidemiologic study found that the total number of SLAP repairs is decreasing while biceps tenodesis is increasing. SLAP repairs were performed for younger patients and biceps tenodeses were performed for older patients. This study demonstrates that clinical practice reflective of recent evidence regarding optimal age for SLAP repair is slow to change. While there is ongoing debate as to the gold standard for the surgical management of SLAP tear lesions, our study confirms that there is an increasing trend among orthopedic surgeons favoring biceps tenodesis which may reflect the increasing literature evidence supporting better clinical outcomes after biceps tenodesis compared to SLAP repair.

## Introduction

Clinical application of arthroscopy for the shoulder joint was reported as early as 1980 [1]. Anterosuperior lesions of the labrum were first characterized using arthroscopy in 1985 by Andrews et al. [2]. Snyder et al. later went on to classify these tears into four types depending on the pattern of lesion and morphology and introduced the term superior labrum anterior to posterior (SLAP) [3,4]. Advancements in diagnostic imaging have contributed to the understanding and identification of SLAP lesions which have paved the way for the expansion of the original classification systems to include seven types and subsequently to 10 types by Maffet et al. and Powell et al., respectively [5,6]. Despite our increasing knowledge of patterns of SLAP lesions, the optimal treatment strategy remains controversial. SLAP type II lesions, the most common type encountered in a clinical setting, are defined as the detachment of the labrum and biceps tendon from the superior aspect of the glenoid [3,6].

Earlier studies have reported arthroscopic SLAP tear repairs for definitive treatment of SLAP type II lesions [7-9]. An epidemiologic study on SLAP repairs in the United States has shown that there was an increase in the incidence of SLAP repairs between 2004 and 2009 [10]. However, overall clinical outcomes have been mixed with conflicting results. Despite studies reporting positive outcomes [11,12], recent literature has reported less than ideal outcomes in certain pathologies and patient demographics thereby scrutinizing the role of arthroscopic repair in treating SLAP tears [13-16]. Furthermore, a three-armed randomized clinical trial comparing SLAP repair, biceps tenodesis, and sham surgery found that neither of the two surgical procedures had a significant benefit over sham surgery in the Norway population [16]. A follow-up randomized clinical trial using the same patients in Schrøder et al. found that the SLAP repair vs. biceps tenodesis trends mean length of return to work following SLAP repair, biceps tenodesis, and sham surgery was not significantly different. In recent studies, biceps tenodesis has been suggested as an alternative surgical technique for management of SLAP tears [17,18]. Boileau et al. compared outcomes after SLAP lesion repair and arthroscopic biceps tenodesis and found return to previous level of sports of 20% and 87% in each respective cohort. Follow-up studies have reported favorable patient-reported outcome (PRO) scores and return-to-play rates in support of biceps tenodesis [19,20]. To evaluate the surgeon preference for repair vs. biceps tenodesis, Patterson et al. queried the American Board of Orthopedic Surgery part II database between 2002 and 2011 and found a downward trend for SLAP repairs (from 69.3% to 44.8%) and an upward trend for biceps tenodesis (from 1.9% to 18.8%). Studies evaluating trends in the use of SLAP repair or biceps tenodesis range from 2001 to 2014 [10,21,22]. However, with the evolving understanding of the pathology and treatment options, there is significant value to qualify and quantify the more current trends in the use of the two surgical techniques for management SLAP tear lesions.

The purpose of this epidemiologic study is to report general trends in the number of SLAP tear repairs and biceps tenodesis performed along with the patient and hospital characteristics within the period of 2016-2018 using encounter data from the National Ambulatory Surgical Sample (NASS).

## Materials and methods

Data source

NASS, Healthcare Cost and Utilization Project (HCUP), and Agency for Healthcare Research and Quality were used as the sources of data for this epidemiologic study. The NASS database contains national estimates of major ambulatory surgery encounters carried out in hospital-owned facilities in the years 2016, 2017, and 2018. HCUP partner organizations across 32-34 states contributed information to the NASS depending on the year. However, these states are geographically dispersed and account for 82-83% of the total United States population.

The NASS reports information on patient characteristics and hospital characteristics. Patient characteristics include: sex, median age at admission, patient location (large central metropolitan, large fringe metropolitan, medium metropolitan, small metropolitan, micropolitan, not metropolitan, or micropolitan), and median household income national quartile ($1-42,999, $43,000-53,999, $54,000-70,999, $71,000 or more). Hospital characteristics include: procedures, total median charges, location of hospital (rural, urban), disposition of patient (routine, transfer to short-term hospital, home healthcare, leave against medical advice, skilled nursing facility, intermediate care, and another type of facility), hospital census region (northeast, midwest, south, west), control/ownership of hospital (public, voluntary, proprietary), and location/teaching status of hospital (rural, urban nonteaching, urban teaching). Current Procedural Terminology (CPT) codes were utilized to identify patients that underwent SLAP repair or biceps tenodesis between 2016 and 2018. To isolate biceps tenodesis that was performed for the treatment of SLAP tears, International Classification of Diseases 10th revision (ICD-10) codes - S43431A, S43432A, and S43439A were used to isolate biceps tenodesis that was performed for a confirmed diagnosis of SLAP tear.

## Results

The national estimates of encounters from the NASS database reported 29,931 SLAP repairs in 2016 and each subsequent year saw a decline to 26,509 repairs in 2017, and 23,451 repairs in 2018 (p<0.0001). Conversely, in 2016 there were 19,221 biceps tenodeses and each subsequent year saw an increase to 21,625 biceps tenodeses in 2017, and 22,867 biceps tenodeses in 2018 (p<0.0001) (Table 1).

**Table 1:** National hospital characteristics for biceps tenodesis vs. SLAP repairs. *P-value by trend analysis. **Dollar in 2018. ***Skilled nursing facility, intermediate care, and another type of facility.

Variables		2016 (N=49,152)	2017 (N=48,134)	2018 (N=46,318)	p-Value
Procedures	Biceps tenodesis	19,221 (39.1%)	21,626 (44.9%)	22,867 (49.4%)	0.0001*
	SLAP repair	29,931 (60.9%)	26,508 (55.1%)	23,451 (50.6%)	
Total charges**	Median (95% CI)	22,558 (21,404 and 23,711)	23,720 (22,785 and 24,653)	25,501 (24,330 and 26,671)	0.0002*
Location of hospital	Rural	8,326 (16.9%)	8,092 (16.8%)	7,753 (16.7%)	0.41*
	Urban	40,826 (83.1%)	40,042 (83.2%)	38,565 (83.3%)	
Disposition of patient	Routine	44,936 (91.4%)	44,127 (91.7%)	43,072 (92.9%)	0.0001
	Transfer to short-term hospital	19 (0.04%)	20 (0.04%)	11 (0.02%)	
	Home healthcare	84 (0.2%)	81 (0.2%)	71 (0.2%)	
	Against medical advice	2 (0.0%)	8 (0.02%)	4 (0.01%)	
	Other transfers***	29 (0.1%)	34 (0.1%)	39 (0.1%)	
	Missing	4,079 (8.3%)	3,862 (8.0%)	3,113 (6.7%)	
Hospital census region	Northeast	8,075 (16.4%)	7,605 (15.8%)	7,547 (16.3%)	0.0001
	Midwest	13,281 (27.0%)	13,192 (27.4%)	12,869 (27.8%)	
	South	18,462 (37.5%)	17,924 (37.2%)	17,538 (37.8%)	
	West	9,333 (18.9%)	9,413 (19.6%)	8,363 (18.1%)	
Control/ownership of hospital	Public	5,650 (11.5%)	5,603 (11.6%)	5,533 (11.9%)	0.0001
	Voluntary	37,633 (76.5%)	36,500 (75.8%)	35,560 (76.8%)	
	Proprietary	5,869 (11.9%)	6,031 (12.5%)	5,225 (11.3%)	
Location/teaching status of hospital	Rural	8,326 (16.9%)	8,092 (16.8%)	7,753 (16.7%)	0.0001
	Urban nonteaching	14,809 (30.1%)	13,144 (27.3%)	12,556 (27.1%)	
	Urban teaching	26,016 (52.9%)	26,898 (55.9%)	26,008 (56.2%)	

The median age at admission for SLAP repair in 2016 was 39.9 years (95% CI: 38.8, 41.0) and decreased thereafter to 39.1 years (95% CI: 38.1, 40.0) in 2017 and 38.6 years (95% CI: 37.5, 39.7) in 2018 (p=0.29) (Table 2).

**Table 2:** Slap repair patient characteristics by years. *P-value by trend analysis.

Variables		2016 (N=29,931)	2017 (N=26,509)	2018 (N=23,451)	p-Value
Sex	Male	20,804 (69.5%)	18,475 (69.7%)	16,144 (68.8%)	0.11*
	Female	9,119 (30.5%)	80,32 (30.3%)	7,308 (31.2%)	
Age at admission	Median (95% CI)	39.9 (38.8, 41.0)	39.1 (38.1, 40.0%)	38.6 (37.5, 39.7)	0.29*
Patient location	Large central metropolitan	7,009 (23.4%)	6,273 (23.7%)	5,347 (22.8%)	0.0001
	Large fringe metropolitan	7,425 (24.8%)	6,441 (24.3%)	5,944 (25.3%)	
	Medium metropolitan	6,407 (21.4%)	5,645 (21.3%)	4,638 (19.8%)	
	Small metropolitan	2,625 (8.8%)	2,341 (8.8%)	2,158 (9.2%)	
	Micropolitan	4,062 (13.6%)	3,572 (13.5%)	3,338 (14.2%)	
	Not metropolitan or micropolitan	2,372 (7.9%)	2,210 (8.3%)	2,012 (8.6%)	
Median household income national quartile for patient ZIP code	$1-42,999	6,257 (20.9%)	5,707 (21.5%)	5,178 (22.1%)	0.0001
	$43,000-53,999	7,771 (25.9%)	7,293 (27.5%)	6,664 (28.4%)	
	$54,000-70,999	7,911 (26.4%)	6,795 (25.6%)	5,874 (25.1%)	
	$71,000 or more	7,497 (25.1%)	6,331 (23.9%)	5,430 (23.2%)	
Primary expected payer	Medicare	2,992 (10.0%)	2,562 (9.7%)	2,413 (10.3%)	0.054
	Medicaid	3,784 (12.6%)	3,554 (13.4%)	3,074 (13.1%)	
	Private including HMO	17,875 (59.7%)	15,639 (59.0%)	13,906 (59.3%)	
	Self-pay	348 (1.2%)	298 (1.1%)	257 (1.1%)	
	No charge	7 (0.02%)	7 (0.03%)	8 (0.03%)	
	Other	4,884 (16.3%)	4,397 (16.6%)	3,747 (16.0%)	

In contrast, the median age at admission for biceps tenodesis in 2016 was 52.2 years (95% CI: 51.8, 52.6) and increased each year to 52.9 years (95% CI: 52.5, 53.3) in 2017, and 53.3 years (95% CI: 52.9, 53.8) in 2018 (p=0.0003) (Table 3). The percentage of males treated with SLAP repair fluctuated with 69.5% in 2016, 69.7% in 2017, and 68.8% in 2018 (p=0.11). Contrary to this, the percentage of males treated with biceps tenodesis decreased from 66.1% in 2016, 65.3% in 2017, and 63.9% in 2018 (p=0.0001).

**Table 3:** Biceps tenodesis patient characteristics by years. *P-value by trend analysis. HMO: Health Maintenance Organization

Variables		2016 (N=19,221)	2017 (N=21,625)	2018 (N=22,867)	p-Value
Sex	Male	12,693 (66.1%)	14,113 (65.3%)	14,619 (63.9%)	0.0001*
	Female	6,521 (33.9%)	7,513 (34.7%)	8,248 (36.1%)	
Age at admission	Median (95% CI)	52.2 (51.8 and 52.6)	52.9 (52.5 and 53.3)	53.3 (52.9 and 53.8)	0.0003*
Patient location	Large central metropolitan	3,529 (18.4%)	3,985 (18.4%)	4,306 (18.8%)	0.0001
	Large fringe metropolitan	5,039 (26.2%)	5,715 (26.4%)	6,099 (26.6%)	
	Medium metropolitan	4,102 (21.3%)	4,846 (22.4%)	5,151 (22.5%)	
	Small metropolitan	2,052 (10.7%)	2,150 (9.9%)	2,196 (9.6%)	
	Micropolitan	2,836 (14.7%)	3,140 (14.5%)	3,355 (14.6%)	
	Not metropolitan or micropolitan	1,648 (8.6%)	1,777 (8.2%)	1,750 (7.7%)	
Median household income national quartile for patient ZIP code	$1-42,999	3,958 (20.6%)	4,189 (19.4%)	4,605 (20.1%)	0.0001
	$43,000-53,999	5,081 (26.4%)	6,139 (28.4%)	6,599 (28.9%)	
	$54,000-70,999	5,261 (27.4%)	5,936 (27.5%)	6,183 (27.0%)	
	$71,000 or more	4,614 (24.0%)	5,059 (23.4%)	5,193 (22.7%)	
Primary expected payer	Medicare	3,431 (17.8%)	3,981 (18.4%)	4,412 (19.3%)	0.0001
	Medicaid	1,737 (9.0%)	1,819 (8.4%)	2,199 (9.6%)	
	Private including HMO	10,667 (55.5%)	11,745 (54.3%)	11,997 (52.5%)	
	Self-pay	207 (1.1%)	251 (1.2%)	283 (1.2%)	
	No charge	7 (0.04%)	13 (0.06%)	11 (0.05%)	
	Other	3,161 (16.5%)	3,773 (17.4%)	3,939 (17.2%)	

Between 2016 and 2018, the total charge for SLAP repair increased by 13.5% from $22,572 to $25,621 (p=0.0006), whereas biceps tenodesis increased 10.0% from $22,482 to $24,739 (p=0.0087) (Table 4). The median total charge between 2016 and 2018 for SLAP repair was $23,839 (95% CI: $23,027 and $24,650) and for biceps tenodesis was $23,734 (95% CI: $22,576 and $24,890) (p=0.33) (Table 5). A large percentage of patients were treated at urban hospitals 83.1% in 2016, 83.2% in 2017, and 3.3% in 2018 (p=0.41). When urban hospitals were stratified by teaching and nonteaching status, there was a 5.4% increase in percentage of SLAP repairs being performed in teaching hospitals (54.0% in 2016, 57.2% in 2017, and 56.9% in 2018) from 2016 to 2018, whereas there was an 8.2% increase in percentage of biceps tenodesis performed in teaching hospitals (51.2% in 2016, 54.3% in 2017, and 55.4% in 2018).

**Table 4:** Slap repair hospital characteristics by years. *Dollar in 2018. **P-value by trend analysis. ***Skilled nursing facility, intermediate care, and another type of facility.

Variables		2016 (N=29,931)	2017 (N=26,509)	2018 (N=23,451)	p-Value
Total charges*	Median (95% CI)	22,572 (21,298, 23,846)	23,688 (22,671, 24,705)	25,621 (24,372, 26,870)	0.0006**
Location of hospital	Rural	5,078 (17.0%)	4,562 (17.2%)	4,143 (17.7%)	0.035**
	Urban	24,853 (83.0%)	21,947 (82.8%)	19,308 (82.3%)	
Disposition of patient	Routine	27,597 (92.2%)	24,449 (92.2%)	21,701 (92.5%)	0.0047
	Transfer to short-term hospital	14 (0.1%)	10 (0.04%)	2 (0.01%)	
	Home healthcare	46 (0.2%)	39 (0.2%)	23 (0.1%)	
	Against medical advice	2 (0.01%)	5 (0.02%)	4 (0.02%)	
	Other transfers***	19 (0.1%)	21 (0.1%)	17 (0.1%)	
	Missing	2,253 (7.5%)	1,984 (7.5%)	1,697 (7.2%)	
Hospital census region	Northeast	5,569 (18.6%)	4,848 (18.3%)	4,416 (18.8%)	0.0001
	Midwest	7,213 (24.1%)	6,562 (24.7%)	5,978 (25.5%)	
	South	11,042 (36.9%)	9,837 (37.1%)	8,991 (38.3%)	
	West	6,107 (20.4%)	5,261 (19.9%)	4,066 (17.3%)	
Control/ownership of hospital	Public	3,143 (10.5%)	2,877 (10.9%)	2,667 (11.4%)	0.0002
	Voluntary	22,866 (76.4%)	20,043 (75.6%)	17,870 (76.2%)	
	Proprietary	3,921 (13.1%)	3,589 (13.5%)	2,913 (12.4%)	
Location/teaching status of hospital	Rural	5,078 (17.0%)	4,562 (17.2%)	4,143 (17.7%)	0.0001
	Urban nonteaching	8,668 (29.0%)	6,785 (25.6%)	5,967 (25.4%)	
	Urban teaching	16,184 (54.0%)	15,162 (57.2%)	13,341 (56.9%)	

**Table 5:** Biceps tenodesis hospital characteristics by years. *Dollar in 2018. **P-value by trend analysis. ***Skilled nursing facility, intermediate care, and another type of facility.

Variables		2016 (N=19,221)	2017 (N=21,625)	2018 (N=22,867)	p-Value
Total charges*	Median (95% CI)	22,482 (20,795, 24,169)	23,933 (21,934, 25,930)	24,739 (22,739, 26,738)	0.0087**
Location of hospital	Rural	3,248 (16.9%)	3,529 (16.3%)	3,610 (15.8%)	0.0021**
	Urban	15,973 (83.1%)	18,096 (83.7%)	19,257 (84.2%)	
Disposition of patient	Routine	17,340 (90.2%)	19,679 (91.0%)	21,371 (93.5%)	0.0001
	Transfer to short-term hospital	4 (0.02%)	10 (0.05%)	9 (0.04%)	
	Home healthcare	39 (0.2%)	42 (0.2%)	48 (0.2%)	
	Against medical advice	0	3 (0.02%)	0	
	Other transfers***	10 (0.1%)	13 (0.1%)	21 (0.1%)	
	Missing	1,826 (9.5%)	1,877 (8.7%)	1,415 (6.2%)	
Hospital census region	Northeast	2,506 (13.0%)	2,757 (12.8%)	3,131 (13.7%)	0.0001
	Midwest	6,068 (31.6%)	6,629 (30.7%)	6,890 (30.1%)	
	South	7,420 (38.6%)	8,087 (37.4%)	8,547 (37.4%)	
	West	3,226 (16.8%)	4,152 (19.2%)	4,297 (18.8%)	
Control/ownership of hospital	Public	2,507 (13.0%)	2,726 (12.6%)	2,865 (12.5%)	0.0001
	Voluntary	14,765 (76.8%)	16,458 (76.1%)	17,689 (77.4%)	
	Proprietary	1,948 (10.1%)	2,442 (11.3%)	2,312 (10.1%)	
Location/teaching status of hospital	Rural	3,248 (16.9%)	3,530 (16.3%)	3,611 (15.8%)	0.0001
	Urban nonteaching	6,140 (31.9%)	6,359 (29.4%)	6,589 (28.8%)	
	Urban teaching	9,833 (51.2%)	11,736 (54.3%)	12,667 (55.4%)	

Hospital census region demonstrated that during the three-year period, the northeast and west regions had higher percentages of SLAP repairs (18.6% and 19.3%, respectively) compared to biceps tenodesis (13.2% and 18.3%, respectively) (Table 6). In comparison, the midwest and south regions reported lower percentages of SLAP repairs (24.7% and 37.4%, respectively) compared to biceps tenodesis (30.7% and 37.8%, respectively).

**Table 6:** Patient characteristics by procedures during 2016-2018. SLAP: superior labrum from anterior to posterior

Variables		Biceps tenodesis (N=63,713)	SLAP repair (N=79,891)	p-Value
Sex	Male	41,424 (65.0%)	55,423 (69.4%)	0.0001
	Female	22,282 (35.0%)	24,459 (30.6%)	
Age at admission	Median (95% CI)	52.8 (52.6, 53.1)	39.2 (38.5, 40.0)	0.0001
Patient location	Large central metropolitan	11,821 (18.5%)	18,628 (23.3%)	0.0001
	Large fringe metropolitan	16,853 (26.4%)	19,810 (24.8%)	
	Medium metropolitan	14,098 (22.1%)	16,690 (20.9%)	
	Small metropolitan	6,398 (10.0%)	7,124 (8.9%)	
	Micropolitan	9,332 (14.6%)	10,974 (13.7%)	
	Not metropolitan or micropolitan	5,175 (8.1%)	6,593 (8.3%)	
Median household income national quartile for patient ZIP code	$1-42,999	12,751 (20.0%)	17,142 (21.5%)	0.0001
	$43,000-53,999	17,819 (28.0%)	21,727 (27.2%)	
	$54,000-70,999	17,381 (27.3%)	20,581 (25.8%)	
	$71,000 or more	14,867 (23.3%)	19,258 (24.1%)	
Primary expected payer	Medicare	11,825 (18.5%)	7,966 (9.9%)	0.0001
	Medicaid	5,756 (9.0%)	10,412 (13.0%)	
	Private including HMO	34,408 (54.0%)	47,421 (59.4%)	
	Self-pay	741 (1.2%)	903 (1.1%)	
	No charge	31 (0.05%)	22 (0.03%)	
	Other	10,874 (17.1%)	13,027 (16.3%)	

## Discussion

This is the first epidemiologic study to report the trends in SLAP repairs and biceps tenodesis using the NASS database for the period of 2016-2018. Of note, this epidemiologic study found that the number of SLAP repairs is decreasing whereas the number of biceps tenodeses is increasing. Furthermore, we found that the median age of patients at admission for SLAP repairs was decreasing albeit not statistically significant compared to the significant increase in median age for biceps tenodeses. In addition, the percentage of males treated with SLAP repair fluctuated with no difference via trend analysis, however, the percentage of males treated with biceps tenodesis decreased significantly over the three-year period. SLAP tears are known to be a cause of shoulder pain, instability, decreasing range of motion, and shoulder dysfunction particularly [20,23]. Surgical management of these lesions is indicated after a trial of conservative therapy. There are two options for this shoulder pathology which involve repair of the SLAP tear or biceps tenodesis, however, there is an ongoing debate within literature on the preferred form of surgical management [17]. A systematic review and meta-analysis by Li et al. found that patient satisfaction with surgery and return to sporting activity SLAP repair vs. biceps tenodesis trends rates were significantly superior in the biceps tenodesis group than in the SLAP repair groups [18]. However, postoperative stiffness and reoperation rates were noted to have no significant 144 differences. A recent systematic review by Civan et al. reported greater patient satisfaction and return to reinjury sports levels and lower revision surgery rates in favor of biceps tenodesis when compared with SLAP repair [10]. The recent systematic reviews and meta-analysis of outcomes after SLAP repair or biceps tenodesis published within the past two years favor the biceps tenodesis [24,25]. The age of the patient undergoing surgical management of SLAP tears has been considered an important factor in choosing the appropriate strategy [24,25]. Our study found that patients undergoing SLAP repair were found to be significantly younger compared to biceps tenodesis. These results validate studies that recommend biceps tenodesis in the older population [12,23] due to reports of persistent pain and stiffness following arthroscopic repair in older patients [13,26,27]. Provencher et al. found that patients greater than 36 years of age were associated with higher rates of failure following SLAP repair and reoperation [24].

Furthermore, biceps tenodesis has been suggested to provide good outcomes and return to pre-injury sports activity when used as a reoperation for failed SLAP repair [17]. Our data revealed that the median age at admission for patients treated with SLAP repair decreased each subsequent year. However, trend analysis did not reveal a statistically significant decrease and the median age at all time points was greater than 35 years which may be indicative of a possible delay from clinical research to widespread change in clinical practice by surgeons. SLAP repair vs. biceps tenodesis trends between 2016 and 2018, the median total charge for both SLAP repair and biceps tenodesis increased. However, biceps tenodesis increased by 22.8% compared to the 13.5% increase for SLAP repair. During this time, inflation could only account for 6.0% of the increase according to the Bureau of Labor Statistics Consumer Price Index inflation calculator. Furthermore, the total charges for biceps tenodesis were less than SLAP repair at all time points. Factors such as operation time, patient co-morbidities, and the volume of surgical facilities have been shown to affect costs for arthroscopic SLAP repair and biceps tenodesis [28]. Notably, female sex was associated with lower overall costs [28]. This study found that biceps tenodesis saw a significant increasing trend in percentage of females compared to SLAP repair which may have contributed to a smaller increase in total charges for biceps tenodesis across the three-year period. We found that there was a small increase in the use of both procedures in teaching hospitals within the three-year period suggesting that local opinion is important and thus the reported regional 176 differences support the impression that treatment strategies are not evidenced based.

Limitations

This study is not without limitations. One of which is the differences in the participating states that contributed information to the NASS. However, as previously stated 32-34 states contributed information depending on the reporting year and represent diverse hospital census regions spanning across the northeast, midwest, south, and west geographies of the United States. Furthermore, we are unable to confirm if the procedures were primary or reoperations to treat previously failed operations. Therefore, the previous limitation could impact the biceps tenodesis group since it is possible that a proportion may be revision surgery for a prior SLAP repair that has failed.

## Conclusions

The results of our epidemiologic study found that the total number of SLAP repairs is decreasing while biceps tenodesis is rising. SLAP repairs were performed largely for younger patients and biceps tenodesis was performed for older patients. This study demonstrates that clinical practice reflective of recent evidence regarding optimal age for SLAP repair is slow to change. While there is ongoing debate as to the gold standard for the surgical management of SLAP tear lesions, our study confirms that there is an increasing trend among orthopedic surgeons favoring biceps tenodesis which may reflect the increasing literature evidence supporting better clinical outcomes.

## References

[1] Gottschalk MB, Karas SG, Ghattas TN, Burdette R (2014). Subpectoral biceps tenodesis for the treatment of type II and IV superior labral anterior and posterior lesions. Am J Sports Med.

[2] Andrews JR, Carson WG Jr, McLeod WD (1985). Glenoid labrum tears related to the long head of the biceps. Am J Sports Med.

[3] Snyder SJ, Banas MP, Karzel RP (1995). An analysis of 140 injuries to the superior glenoid labrum. J Shoulder Elbow Surg.

[4] Alpert JM, Wuerz TH, O'Donnell TF, Carroll KM, Brucker NN, Gill TJ (2010). The effect of age on the outcomes of arthroscopic repair of type II superior labral anterior and posterior lesions. Am J Sports Med.

[5] Maffet MW, Gartsman GM, Moseley B (1995). Superior labrum-biceps tendon complex lesions of the shoulder. Am J Sports Med.

[6] Powell SE, Nord KD, Ryu RK (2012). The diagnosis, classification, and treatment of SLAP lesions. Oper Tech Sports Med.

[7] Johnson LL (1980). Arthroscopy of the shoulder. Orthop Clin North Am.

[8] Brockmeier SF, Voos JE, Williams RJ 3rd, Altchek DW, Cordasco FA, Allen AA (2009). Outcomes after arthroscopic repair of type-II SLAP lesions. J Bone Joint Surg Am.

[9] Burkhart S, Morgan C (1998). The peel-back mechanism: its role in producing and extending posterior type II SLAP lesions and its effect on SLAP repair rehabilitation. Arthroscopy.

[10] Civan O, Bilsel K, Kapicioglu M, Ozenci AM (2021). Repair versus biceps tenodesis for the slap tears: a systematic review. J Orthop Surg (Hong Kong).

[11] Ek ET, Shi LL, Tompson JD, Freehill MT, Warner JJ (2014). Surgical treatment of isolated type II superior labrum anterior-posterior (SLAP) lesions: repair versus biceps tenodesis. J Shoulder Elbow Surg.

[12] Erickson BJ, Jain A, Abrams GD, Nicholson GP, Cole BJ, Romeo AA, Verma NN (2016). SLAP lesions: trends in treatment. Arthroscopy.

[13] Katz LM, Hsu S, Miller SL, Richmond JC, Khetia E, Kohli N, Curtis AS (2009). Poor outcomes after SLAP repair: descriptive analysis and prognosis. Arthroscopy.

[14] Kim SH, Ha KI, Kim SH, Choi HJ (2002). Results of arthroscopic treatment of superior labral lesions. J Bone Joint Surg Am.

[15] Kim SJ, Lee IS, Kim SH, Woo CM, Chun YM (2012). Arthroscopic repair of concomitant type II SLAP lesions in large to massive rotator cuff tears: comparison with biceps tenotomy. Am J Sports Med.

[16] Kim TK, Queale WS, Cosgarea AJ, McFarland EG (2003). Clinical features of the different types of SLAP lesions: an analysis of one hundred and thirty-nine cases. J Bone Joint Surg Am.

[17] Schrøder CP, Skare Ø, Reikerås O, Mowinckel P, Brox JI (2017). Sham surgery versus labral repair or biceps tenodesis for type II SLAP lesions of the shoulder: a three-armed randomised clinical trial. Br J Sports Med.

[18] Li LT, Chuck C, Bokshan SL, DeFroda SF, Owens BD (2021). Cost comparison of open and arthroscopic treatment options for SLAP tears. Arthrosc Sports Med Rehabil.

[19] Boileau P, Parratte S, Chuinard C, Roussanne Y, Shia D, Bicknell R (2009). Arthroscopic treatment of isolated type II SLAP lesions: biceps tenodesis as an alternative to reinsertion. Am J Sports Med.

[20] Li M, Shaikh AB, Sun J, Shang P, Shang X (2018). Effectiveness of biceps tenodesis versus SLAP repair for surgical treatment of isolated SLAP lesions: a systemic review and meta-analysis. J Orthop Translat.

[21] Morgan CD, Burkhart SS, Palmeri M, Gillespie M (1998). Type II SLAP lesions: three subtypes and their relationships to superior instability and rotator cuff tears. Arthroscopy.

[22] Pagnani MJ, Speer KP, Altchek DW, Warren RF, Dines DM (1995). Arthroscopic fixation of superior labral lesions using a biodegradable implant: a preliminary report. Arthroscopy.

[23] Patterson BM, Creighton RA, Spang JT, Roberson JR, Kamath GV (2014). Surgical trends in the treatment of superior labrum anterior and posterior lesions of the shoulder: analysis of data from the American Board of Orthopaedic Surgery Certification Examination Database. Am J Sports Med.

[24] Paxinos A, Walton J, Rütten S, Müller M, Murrell GA (2006). Arthroscopic stabilization of superior labral (SLAP) tears with biodegradable tack: outcomes to 2 years. Arthroscopy.

[25] Pogorzelski J, Horan MP, Hussain ZB, Vap A, Fritz EM, Millett PJ (2018). Subpectoral biceps tenodesis for treatment of isolated type II SLAP lesions in a young and active population. Arthroscopy.

[26] Provencher MT, McCormick F, Dewing C, McIntire S, Solomon D (2013). A prospective analysis of 179 type 2 superior labrum anterior and posterior repairs: outcomes and factors associated with success and failure. Am J Sports Med.

[27] Snyder SJ, Karzel RP, Pizzo WD, Ferkel RD, Friedman MJ (1990). SLAP lesions of the shoulder. Arthroscopy.

[28] Zhang AL, Kreulen C, Ngo SS, Hame SL, Wang JC, Gamradt SC (2012). Demographic trends in arthroscopic SLAP repair in the United States. Am J Sports Med.

